# Sodium Selenite Attenuates Monocrotaline-Induced Pulmonary Arterial Hypertension by Upregulating GPX3 and Ameliorating Vascular Remodeling

**DOI:** 10.33549/physiolres.935734

**Published:** 2026-04-01

**Authors:** Zihua LIU, Xue LIU, Richard MPRAH, Hong SUN, Joseph ADU-AMANKWAAH, Xiuhua PAN, Shouquan CHENG, Bing XIE, Cheng WANG, Weili QIAO

**Affiliations:** 1 Department of Physiology, Xuzhou Medical University, Xuzhou, Jiangsu, China; 2 Department of Cardiology, Affiliated Hospital of Xuzhou Medical University, Xuzhou, Jiangsu, China

**Keywords:** Sodium selenite, Pulmonary arterial hypertension, Glutathione peroxidase 3, Oxidative stress

## Abstract

Selenium deficiency is implicated in pulmonary arterial hypertension (PAH). This study investigated the effects of sodium selenite (SSE) on PAH and its underlying mechanisms. In a monocrotaline-induced PAH rat model, SSE treatment significantly improved right ventricular systolic pressure (RVSP), mean pulmonary arterial pressure (mPAP), and vascular remodeling. It reduced collagen deposition and partially restored the expression of Collagen 1 and SM22α. SSE upregulated glutathione peroxidase 3 (GPX3) expression and attenuated oxidative stress, as evidenced by enhanced SOD activity and decreased MDA levels. In vitro, SSE inhibited H_2_O_2_ and angiotensin II-induced proliferation, oxidative stress, and phenotypic switching in pulmonary artery smooth muscle cells (PASMCs), while upregulating GPX3 at both mRNA and protein levels. These findings demonstrate that SSE alleviates PAH by enhancing GPX3-mediated antioxidant defense and suppressing vascular remodeling, supporting its potential as a therapeutic strategy for PAH.

## Introduction

Pulmonary arterial hypertension (PAH) remains a devastating cardiovascular disorder characterized by progressive pulmonary vascular remodeling, elevated pulmonary vascular resistance, and eventual right heart failure [1–3]. Pulmonary vascular remodeling is characterized by abnormal proliferation of pulmonary artery smooth muscle cells (PASMCs), which is considered to be the major cause for the development of PAH [4,5]. Despite advances in targeted therapies such as endothelin receptor antagonists and phosphodiesterase-5 inhibitors, the long-term prognosis for PAH patients remains poor, with a 5-year survival rate of approximately 60 % [6–9]. This underscores the urgent need to explore therapeutic strategies.

Oxidative stress has emerged as a central driver in PAH pathogenesis, contributing to endothelial dysfunction, vascular smooth muscle cell hyperplasia, and extracellular matrix deposition [10–12]. Excessive reactive oxygen species (ROS) production disrupts redox signaling, thereby promoting vascular smooth muscle cell proliferation, migration, and resistance to apoptosis—key hallmarks of pulmonary vascular remodeling [13]. While antioxidant-based therapies (e.g., superoxide dismutases, tocopherols, and flavonoids) have shown promise in preclinical PAH studies, their clinical efficacy remains inconsistent [11,14]. This inconsistency likely stems from the complex pathophysiology of PAH and the significant heterogeneity among patients. Consequently, targeting oxidative stress pathways continues to be an actively explored therapeutic frontier for PAH.

Selenium, an essential trace element, serves as a critical component of key antioxidant enzymes and plays a central role in maintaining redox homeostasis [15,16]. In recent years, growing attention has been drawn to the association between selenium deficiency and the pathogenesis of PAH [17]. Epidemiological studies have consistently shown that serum selenium levels are significantly decreased in PAH patients and inversely correlate with disease severity [18,19]. These observations suggest that selenium may influence the pathogenesis of PAH by modulating the activity of antioxidant enzymes. The selenoprotein family serves as the primary vehicle for selenium’s antioxidant functions in biological systems, with the glutathione peroxidase (GPX) family being particularly important [20]. Among the GPX family, glutathione peroxidase 3 (GPX3)—the only extracellular secreted isoform—acts as a vital scavenger of ROS [21–24] and is strongly implicated in vascular oxidative stress and endothelial dysfunction [25]. Given its pivotal role in antioxidant defense, modulating the expression of GPX3 may offer a novel strategy for alleviating oxidative stress and ameliorating pathological features in PAH.

Sodium selenite (SSE), a common bioavailable form of selenium supplementation, has been shown to enhance the expression and activity of selenoproteins. A previous study has suggested that SSE ameliorates pulmonary vascular remodeling and right ventricular dysfunction in PAH rats [26]. Exploring the regulatory effect of SSE on GPX3 expression and its potential therapeutic application in PAH is therefore of significant interest.

This research explores the impact of SSE on PAH and elucidates its underlying mechanisms. In vivo, we systematically assessed the effects of SSE on hemodynamics, vascular remodeling, oxidative stress and GPX3 distribution in a monocrotaline (MCT)-induced PAH rat model. In vitro, we explored how SSE regulates GPX3 expression and its anti-proliferative and antioxidant effects in PASMCs exposed to H_2_O_2_ and angiotensin II (Ang II). The study aim to provide experimental evidence supporting the potential therapeutic application of selenium in PAH.

## Methods

### Ethical approval and animal care

This study was conducted in accordance with the ethical guidelines for the care and use of laboratory animals. The protocol was approved by the Laboratory Animal Ethics Committee of Xuzhou Medical University (Approval No. 202403T012). Male Sprague-Dawley rats (180–220 g) from the Laboratory Animal Center of Xuzhou Medical University (Breeding License No. SCXK (Su) 2020-0011) were housed under controlled conditions (temperature: 20–26 °C; humidity: 50±5 %; 12-hour light/dark cycle) with free access to food and water.

## Materials and Instruments

Materials: MCT (Macklin C804263); Sodium Selenite (MCE HY-W686381); Ang II (MCE HY-13948); GPX3 (Proteintech 13947-1-AP); Collagen I (COL1) (Biodragon BD-PM3764); SM22α (Proteintech 10493-1-AP); β-actin (Proteintech 66009-1-Ig); GPX3 ELISA Kit (Jonlnbio JL34429-96T); HRP-conjugated Goat Anti-Rabbit IgG (Wanleibio WLA023a); HRP-conjugated Rabbit Anti-Mouse IgG (Wanleibio WLA024a); iFluor™ 488 Conjugated Goat anti-rabbit IgG polyclonal Antibody (HUABIO HA1121); iFluor™ 594 Conjugated Goat anti-mouse IgG polyclonal Antibody (HUABIO HA1126); superoxide dismutase (SOD) Activity Assay Kit (Solarbio BC5165); malondialdehyde (MDA) Content Assay Kit (Solarbio BC0025); Reactive Oxygen Species Assay Kit (Solarbio CA1410); Reverse Transcription Kit (Vazyme R223-01); SYBR Green PCR Kit (Vazyme Q711-02). Pressure transducer and multi-channel physiological recorder (Power Lab System, ADInstruments).

### PAH rat model and treatment procedures

The PAH rat model was established by a single intraperitoneal injection of monocrotaline (MCT, 60 mg/kg), following an established protocol that reliably induces hemodynamic alterations characteristic and pulmonary vascular remodeling of PAH within 3–4 weeks in our previous study [27]. Rats in the SSE treatment group received daily SSE treatment at 0.2 mg/kg *via* intragastric administration (gavage) starting from day 3 post-MCT injection for 28 days [17,26,28]. The control and PAH groups received an equivalent volume of normal saline *via* the same route. After right heart and pulmonary function assessments, the rats were euthanized on day 28, and samples were collected for further analysis.

### Cell culture

Rat PASMCs were purchased from Haixing Biotechnology Co., Ltd. and cultured in DMEM supplemented with 10 % fetal bovine serum at 37 °C in a humidified 5 % CO_2_ incubator. Cells were seeded at approximately 70–80 % confluence and allowed to adhere for 24 hours prior to treatment for 36 hours. Model groups were treated with 100 nM H_2_O_2_ or 1 μM Ang II. Treatment groups were co-treated with 100 nM H_2_O_2_ and 2 μM SSE or 1 μM Ang II and 2 μM SSE.

### Right heart catheterization and pulmonary artery pressure measurement

Rats were anesthetized with 1 % tribromoethanol (200 mg/kg, i.p.). Right ventricular function was assessed by cannulating the right ventricle via the right external jugular vein. Pulmonary artery function was evaluated after successful intubation. All data were acquired and analyzed using the Power Lab system.

### Hematoxylin and Eosin (HE) staining

Tissue samples were fixed in 4 % formaldehyde, dehydrated, cleared, paraffin-infiltrated, embedded, and sectioned. Sections were dewaxed and stained with HE. Pulmonary artery wall thickness was measured directly using ImageJ software. For intrapulmonary arterioles, wall thickness percentage (WT %) was calculated as (wall thickness/external diameter) × 100 %, and wall area percentage (WA %) was calculated as (wall area/total vessel area) × 100 %.

### Masson’s trichrome staining

Tissue sections were dewaxed, stained with Masson’s trichrome, and sealed with neutral resin. Six random fields per section were examined under a light microscope for quantitative analysis. The degree of myocardial and vascular fibrosis was assessed using ImageJ Pro Plus 6.0, with collagen volume fraction (CVF %) calculated as (collagen fiber area / total tissue area) × 100 %.

### Immunohistochemical staining

Sections were dewaxed, rehydrated, and treated with 3 % H_2_O_2_ to block endogenous peroxidase activity. After blocking, sections were incubated overnight at 4 °C with primary antibodies against GPX3 (1:300), COL1 (1:200), and SM22α (1:200). Following incubation with secondary antibodies, antigen detection was performed using DAB chromogen, and sections were counterstained with hematoxylin, dehydrated, cleared, and mounted. Six random fields per section were imaged, and positive staining was quantified using ImageJ Pro Plus 6.0. The average optical density (AOD) was calculated as the ratio of positive area optical density to total tissue area.

### Cell viability assay

Cells were seeded in a 96-well plate and incubated overnight at 37 °C in a 5 % CO_2_ atmosphere. Following attachment, cells were treated with test agents, including appropriate controls. After 36 hours of treatment, 10 μL of CCK-8 reagent was added to each well, and the plate was incubated for an additional 2 hours at 37 °C. Absorbance was measured at 450 nm using a microplate reader. Cell viability was expressed as a percentage relative to untreated controls.

### Cell proliferation assay

Cells were fixed with 4 % paraformaldehyde and permeabilized with 0.1 % Triton X-100. According to the manufacturer’s instructions, cells were labeled with EdU, counterstained with Hoechst, and imaged by fluorescence microscopy. Six random fields were captured, and the percentage of EdU-positive cells was quantified using ImageJ Pro Plus 6.0. The proportion of EdU-positive cells was calculated as an indicator of proliferative activity. The average of the six fields was used as the representative value for each experimental group.

### Immunofluorescence

Cells were fixed with 4 % paraformaldehyde, permeabilized with 0.1 % Triton X-100, and blocked with 5 % BSA. Primary antibodies (SM22α 1:100, COL1 1:100, GPX3 1:300) were incubated overnight at 4 °C. After washing, cells were incubated with secondary antibodies and DAPI for nuclei staining. Images from six random fields per sample were captured on a fluorescence microscope and quantified using ImageJ Pro Plus 6.0.

### Western blotting

Proteins were extracted, quantified, and separated by SDS-PAGE. They were transferred to a PVDF membrane, blocked, and incubated with GPX3 primary antibody (1:1000) overnight at 4 °C. After secondary antibody incubation, the blots were imaged with a Tanon-4200 system and analyzed using ImageJ Pro Plus 6.0.

### ELISA for serum GPX3

GPX3 protein levels in rat serum were determined using a commercial Rat GPX3 ELISA Kit (Jonlnbio JL34429-96T) according to the manufacturer’s instructions. The assay was performed by incubating samples and reagents as directed, and absorbance was measured at 450 nm. GPX3 concentrations were interpolated from the standard curve.

### Real-time quantitative PCR (RT-qPCR)

Total RNA extracted from cells using Trizol reagent was quantified and assessed for purity *via* spectrophotometry. cDNA was synthesized from 1 μg RNA using HiScript III RT SuperMix for qPCR (Vazyme R223-01) with a Reverse Transcription Kit. qPCR was conducted using a SYBR Green PCR Kit on a real-time PCR system, with GPX3 expression normalized to the housekeeping gene β-actin. Primers used were: GPX3 forward 5′-GGCATACCAATTATGCGCTGG-3′ and reverse 5′-GTGGGGTAGGGCATCAGTT-3′; β-actin forward 5′-TGAGAGGGAAATCGTGCGTGAC-3′ and reverse 5′-GCTCGTTGCCAATAGTGATGACC-3′. Relative expression levels were determined by the 2^(−Δ ΔCt) method.

### Measurement of oxidative stress index

SOD activity and MDA content were determined according to the manufacturer’s instructions. For ROS detection, cells were incubated with 10 μM DCFH-DA at 37 °C for 30 minutes, followed by washing with PBS. Six random fields per sample were imaged and quantified using a fluorescence microscope and ImageJ Pro Plus 6.0.

### Statistical analysis

Data were analyzed using GraphPad Prism (version 9.0). Quantitative data are presented as mean ± SEM. One-way analysis of variance (ANOVA) was used for comparisons among multiple groups, and post hoc analysis was done using Tukey’s multiple comparisons test. A *p*-value of less than 0.05 was considered statistically significant.

## Results

### Sodium selenite improves hemodynamics in PAH rats

**Fig. 1:**
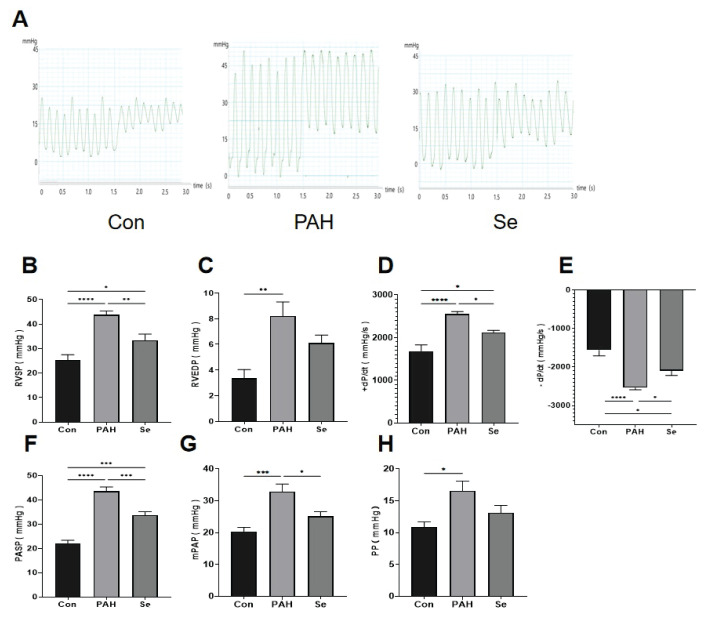
Sodium selenite improves hemodynamics in PAH rats. (**A**) Representative pressure traces from right heart catheterization. (**B**) Right ventricular systolic pressure (RVSP). (**C**) Right ventricular end-diastolic pressure (RVEDP). (**D**) Maximal rate of pressure rise (+dP/dt). (**E**) Maximal rate of pressure decline (−dP/dt). (**F**) Pulmonary artery systolic pressure (PASP). (**G**) Mean pulmonary artery pressure (mPAP). (**H**) Pulse pressure (PP). Data are presented as the mean ± SEM; n=6. *p < 0.05, **p < 0.01, *** *p* < 0.001, ****p < 0.0001 indicate significant differences. Con, control; PAH, pulmonary arterial hypertension; Se, sodium selenite.

In the PAH group, right ventricular systolic pressure (RVSP), end-diastolic pressure (RVEDP), pulmonary arterial systolic pressure (PASP), and mean pulmonary arterial pressure (mPAP) were significantly elevated compared to controls (Fig. 1A–H). Treatment with SSE significantly reduced the elevated pressures (Fig. 1A–H).

### SSE attenuates pulmonary vascular remodeling and fibrosis in PAH rats

**Fig. 2:**
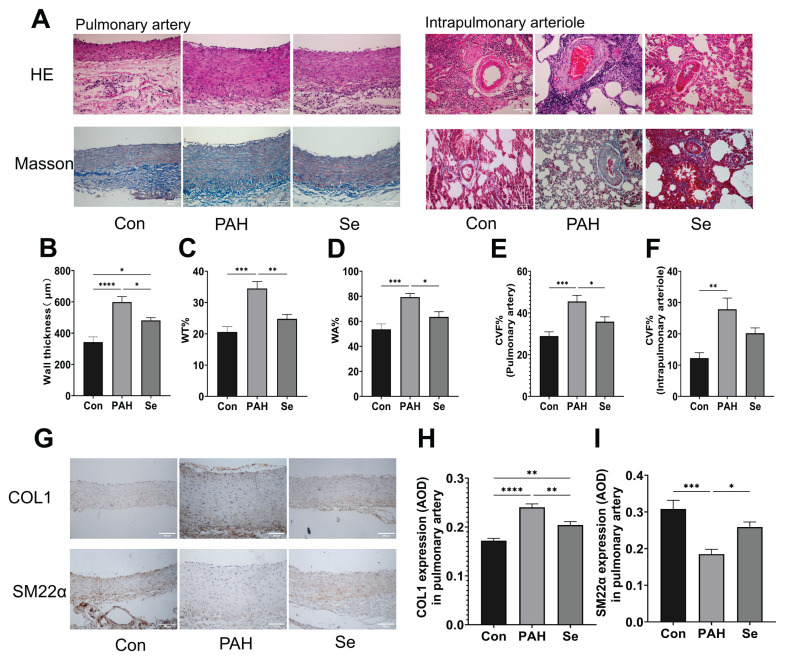
SSE attenuates pulmonary vascular remodeling and fibrosis in PAH rats. (**A**) Representative images of Hematoxylin and Eosin (HE) and Masson’s trichrome staining of pulmonary arteries and intrapulmonary arterioles. Scale bars, 200 μm. (**B**) Wall thickness of pulmonary arteries. (**C**) Wall thickness percentage (WT%) of intrapulmonary arterioles. (**D**) Wall area percentage (WA%) of intrapulmonary arterioles. (**E**) Collagen volume fraction (CVF%) in pulmonary arteries. (**F**) CVF% in intrapulmonary arterioles. (**G**) Representative immunohistochemical staining images for COL1 and SM22α in pulmonary arteries. Brown color indicates positive staining. Scale bar, 200 μm. (**H**) Quantification of COL1 expression (mean optical density, AOD). (**I**) Quantification of SM22α expression (AOD). Data are presented as the mean ± SEM; n=6. *p < 0.05, **p < 0.01, ***p < 0.001, ****p < 0.0001 indicate significant differences. Con, control; PAH, pulmonary arterial hypertension; Se, sodium selenite.

In the PAH rat model, severe vascular structural disruption and collagen deposition were observed. The wall thickness of pulmonary arteries increased, and the WT % and WA % of intrapulmonary arterioles were elevated. The CVF % also significantly increased (Fig. 2A–D, E–F). Additionally, the expression of COL1 was upregulated, while that of SM22α was downregulated in the pulmonary vessels (Fig. 2G–I). These pathological changes were significantly mitigated by SSE treatment.

### SSE upregulates GPX3 expression and alleviates oxidative stress

**Fig. 3:**
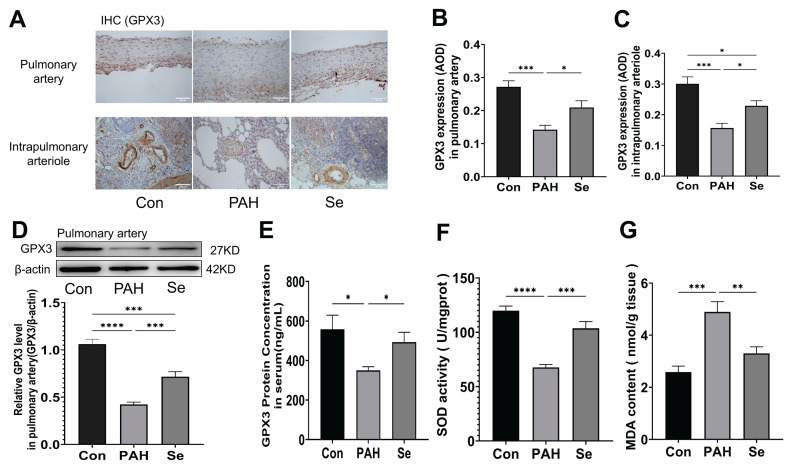
SSE upregulates GPX3 expression and alleviates oxidative stress in PAH rats. (**A**) Representative immunohistochemical staining for GPX3 in pulmonary arteries and intrapulmonary arterioles. Brown areas indicate positive staining. Scale bar, 200 μm. (**B**) Quantification of GPX3 expression (mean optical density, AOD) in pulmonary arteries. (**C**) Quantification of GPX3 expression (AOD) in intrapulmonary arterioles. (**D**) Western blot analysis of GPX3 protein levels in pulmonary artery tissues (loading control: β-actin). Quantification is shown below the blots. (**E**) GPX3 concentration in serum as determined by ELISA. (**F**) SOD activity in lung tissues. (**G**) MDA content in lung tissues. Data are presented as the mean ± SEM; n=6. *p < 0.05, **p < 0.01, ***p < 0.001, ****p < 0.0001 indicate significant differences. Con, control; PAH, pulmonary arterial hypertension; Se, sodium selenite.

In the PAH rat model, GPX3 expression was significantly downregulated in pulmonary arteries and intrapulmonary arterioles, as evidenced by reduced immunostaining intensity (Fig. 3A–C) and decreased protein levels in pulmonary artery tissues (Fig. 3D). Consistently, GPX3 concentration in serum was significantly decreased in PAH rats as measured by ELISA, and SSE treatment significantly increased serum GPX3 concentration (Fig. 3E). Additionally, PAH rats exhibited decreased SOD activity and increased MDA content, which were significantly improved by SSE treatment (Fig. 3F–G).

### SSE inhibits abnormal proliferation and phenotypic switching of PASMCs

**Fig. 4:**
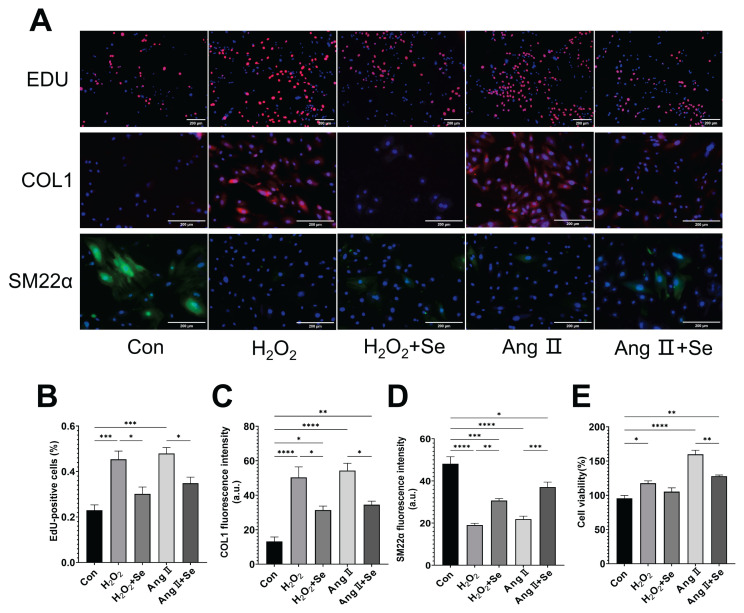
SSE inhibits abnormal proliferation and phenotypic switching of PASMCs in vitro. (**A**) Representative immunofluorescence images. For EdU assay: Red, EdU; Blue, Hoechst (nuclei). For protein staining (COL1 and SM22α): Green/Red, target protein; Blue, DAPI (nuclei). Scale bars, 200 μm. (**B**) Percentage of EdU-positive cells. (**C**) Fluorescence intensity of COL1. (**D**) Fluorescence intensity of SM22α. (**E**) Cell viability percentage measured by CCK-8 assay. Data are presented as the mean ± SEM; n=4. *p < 0.05, **p < 0.01, ***p < 0.001, ****p < 0.0001 indicate significant differences. Con, control; H_2_O_2_, Hydrogen Peroxide; Ang II, Angiotensin II; Se, sodium selenite.

H_2_O_2_ and Ang II induce abnormal proliferation in PASMCs, as evidenced by increased EdU incorporation (Fig. 4A, B) and enhanced cell viability (Fig. 4E). Additionally, H_2_O_2_ and Ang II trigger phenotypic switching in PASMCs, characterized by upregulation of the synthetic phenotype marker COL1 and downregulation of the contractile marker SM22α (Fig. 4A, C and D), indicating an abnormal shift towards a synthetic phenotype. These changes are significantly mitigated by SSE treatment.

### SSE reduces oxidative stress in PASMCs

**Fig. 5:**
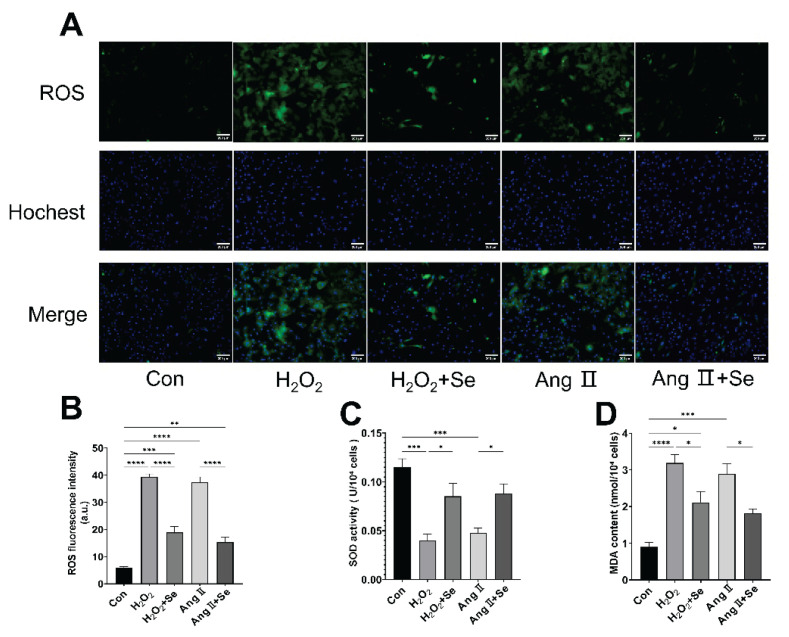
SSE reduces oxidative stress in PASMCs. (**A**) represent-tative immunofluorescence images showing intracellular ROS (green) and Hoechst nuclear staining (blue). (**B**) Quantification of ROS fluorescence intensity. (**C**) SOD activity. (**D**) MDA content. Data are presented as the mean ± SEM; n=4. *p < 0.05, **p < 0.01, ***p < 0.001, ****p < 0.0001 indicate significant differences. Con, control; H_2_O_2_, Hydrogen Peroxide; Ang II, Angiotensin II; Se, sodium selenite.

In PASMCs stimulated by H_2_O_2_ and Ang II, ROS fluorescence intensity was significantly increased (Fig. 5B), indicating heightened oxidative stress. SSE co-treatment markedly reduced this increase (Fig. 5B). Consistent with in vivo findings, SSE increased SOD activity (Fig. 5C) and decreased MDA content (Fig. 5D) in PASMCs under these stress conditions, demonstrating its antioxidant efficacy at the cellular level.

### SSE Upregulates GPX3 Expression in PASMCs

**Fig. 6:**
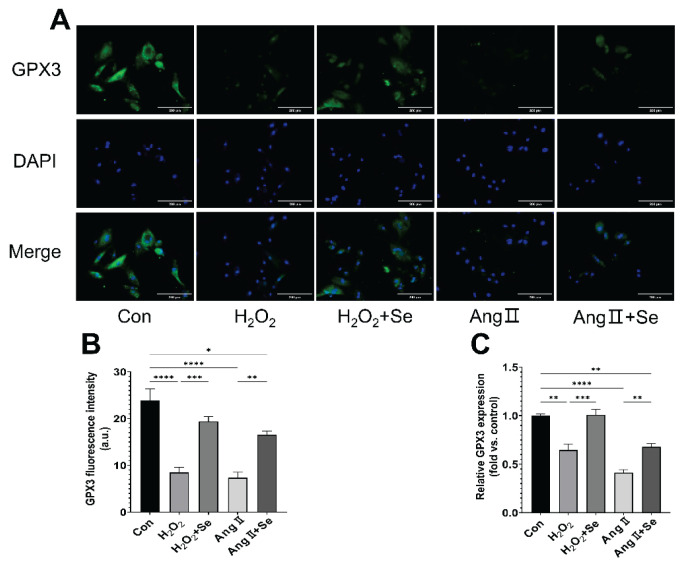
SSE upregulates GPX3 expression in PASMCs under oxidative stress. (**A**) Representative immunofluorescence images showing GPX3 (green), DAPI (blue), and merged channels. Scale bars, 200 μm. (**B**) Quantification of GPX3 fluorescence intensity. (**C**) Relative GPX3 mRNA expression levels normalized to β-actin. Data are presented as the mean ± SEM; n=4. *p < 0.05, **p < 0.01, ***p < 0.001, ****p < 0.0001 indicate significant differences. Con, control; H_2_O_2_, Hydrogen Peroxide; Ang II, Angiotensin II; Se, sodium selenite.

GPX3 expression was decreased in PASMCs treated with H_2_O_2_ and Ang II, as shown by reduced fluorescence intensity and quantitative analysis (Fig. 6A, B). SSE treatment significantly elevated GPX3 expression. Consistent with these findings, GPX3 mRNA expression was downregulated under oxidative stress and upregulated by SSE treatment (Fig. 6C).

## Discussion

This study demonstrates that SSE alleviates experimental PAH by targeting oxidative stress and vascular remodeling, with GPX3 identified as a key mechanistic mediator. In an MCT-induced rat model, treatment with SSE (0.2 mg/kg/day) significantly improved hemodynamics—reducing RVSP and mPAP—and attenuated structural pathology, including pulmonary arterial wall thickening and collagen deposition. At the cellular level, SSE inhibited oxidative stress, prevented the pathological phenotypic switching of PASMCs, and suppressed their aberrant proliferation, effects consistently associated with the upregulation of GPX3. These findings not only confirm the therapeutic potential of SSE but also delineate a specific GPX3-centered pathway. It is noteworthy that while epidemiological studies have linked low selenium levels to PAH severity [18,19], the causal direction—whether deficiency drives pathology or results from it—has been unclear. Our interventional evidence that SSE ameliorates experimental PAH supports a contributory role of selenium deficiency in disease progression.

This study confirms that the therapeutic efficacy of SSE against PAH likely stems from its modulation of the selenoprotein system. Given that the biological functions of selenium are primarily mediated through its incorporation into the selenoprotein family [29,30], our research naturally focused on identifying the key effector molecule within this system. Complementing this line of inquiry, a recent study by Liang *et al.* showed that selenomethionine (an organic form) alleviated PAH by upregulating SELENBP1, primarily affecting fibroblast activation [17]. Our experimental data consistently pointed to GPX3—a crucial secreted antioxidant enzyme in vascular oxidative stress[31,32]. Here, we identify a distinct mechanism for the inorganic compound SSE, previously shown to mitigate vascular remodeling [26], demonstrating that its beneficial effects are mediated through the prominent upregulation of GPX3. The results indicate that different selenium species can exert their effects by regulating selenoproteins. The therapeutic differences among various selenium species require further investigation.

In our experimental model, SSE treatment reversed the downregulation of GPX3 in pulmonary arteries and elevated its concentration in the serum. This restoration of GPX3, a secreted selenoprotein crucial for scavenging extracellular ROS and protecting vascular integrity [25,33], was accompanied by enhanced systemic antioxidant capacity (increased SOD activity, decreased MDA levels). Thus, GPX3 upregulation represents a pivotal mechanism through which SSE counteracts oxidative stress, a central driver of PAH pathogenesis [11]. It is important to note that selenium exhibits a U-shaped dose-response relationship [34]. The dose used here (0.2 mg/kg/day) was based on previous preclinical studies showing efficacy without toxicity [17,26,28]; however, the optimal therapeutic window for PAH warrants further definition.

The alleviation of oxidative stress via GPX3 upregulation contributes to the inhibition of pathogenic vascular cell processes, which underlies the observed amelioration of vascular remodeling. Critically, our in vitro experiments demonstrated that SSE treatment effectively suppressed the aberrant proliferation of PASMCs induced by H_2_O_2_ and Ang II, as measured by EdU incorporation and CCK-8 assay. More importantly, SSE prevented the pathological phenotypic switch of PASMCs: it reversed the downregulation of the contractile marker SM22α and suppressed the upregulation of the synthetic/profibrotic marker collagen I (COL1) triggered by oxidative and pro-inflammatory stimuli. These cellular actions were consistently associated with a marked SSE-induced upregulation of GPX3 expression at both the mRNA and protein levels in stressed PASMCs. Thus, by boosting GPX3, SSE mitigates the oxidative milieu that activates pathological PASMC behavior—specifically, hyperproliferation and the loss of contractile phenotype—thereby addressing a fundamental cellular driver of vascular remodeling.

In summary, our results confirm that SSE is an effective selenium form for mitigating experimental PAH, primarily through a mechanism involving the upregulation of GPX3. This pathway connects the correction of selenium deficiency to the amelioration of oxidative stress and the subsequent inhibition of pathological vascular remodeling. These insights strengthen the rationale for considering selenium status and selenoprotein-targeted strategies in PAH management. While GPX3 is a key mediator identified here, as an efficient selenium donor, SSE may also influence the activity of other selenoproteins within the biological network, a possibility that merits future investigation.

## Limitations and Future Directions

While our study identifies GPX3 upregulation as a central mechanism for SSE’s benefits in experimental PAH, several limitations and future directions warrant consideration to advance its translational potential. First, the therapeutic window of SSE requires precise definition. Selenium exhibits a U-shaped dose-response relationship, wherein both deficiency and excess are harmful [34]. Although the dose used here (0.2 mg/kg/day) was effective and safe in our rodent model, determining the optimal dosage and treatment duration for potential human application necessitates systematic dose-ranging and long-term toxicity studies. Second, the translational efficacy of different selenium forms merits direct comparison. This study focused on the inorganic compound SSE, while other work has employed organic forms like selenomethionine [17]. Future preclinical studies should directly compare the efficacy, safety, and pharmacokinetic profiles of various selenium formulations (e.g., SSE, selenomethionine, selenium-enriched yeast) in PAH models to inform the choice of agent for clinical trials Third, the role of selenoproteins beyond GPX3 should be explored. Selenium’s biological effects are mediated by a network of selenoproteins. Although GPX3 emerged as a key mediator in our setting, it remains possible that SSE’s benefits involve the modulation of other selenoproteins. Future investigations using proteomic or genetic approaches could elucidate the broader selenoprotein landscape influenced by SSE in PAH. Fourth, the potential interplay between SSE/GPX3 and established pathogenic pathways needs examination. Given the central role of pathways such as TGF-β/SMAD signaling in PAH-associated vascular remodeling [35,36], it would be valuable to investigate whether and how SSE-mediated GPX3 upregulation and oxidative stress mitigation interact with these critical signaling cascades.
